# The impact of comorbidities on tuberculosis treatment outcomes in Poland: a national cohort study

**DOI:** 10.3389/fpubh.2023.1253615

**Published:** 2023-09-05

**Authors:** Adam Nowiński, Stefan Wesołowski, Maria Korzeniewska-Koseła

**Affiliations:** ^1^Department of Tuberculosis Epidemiology and Surveillance, National Tuberculosis and Lung Diseases Research Institute, Warsaw, Poland; ^2^National Tuberculosis and Lung Diseases Research Institute, Warsaw, Poland

**Keywords:** mortality, treatment outcome, Poland, tuberculosis, comorbidity

## Abstract

**Background:**

Tuberculosis (TB) is a complex disease associated with other medical conditions, that may affect disease severity. This study aimed to investigate the impact of comorbidities on treatment outcomes and mortality rates in patients with TB in Poland.

**Methods:**

We analyzed a national cohort of 19,217 adult TB patients diagnosed between 2011 and 2016 in Poland. We compared treatment success rates and mortality rates in patients with comorbidities and those without to assess the impact of various comorbidities on these outcomes. Odds ratios (OR) were calculated to quantify the association between comorbidities and TB treatment outcomes.

**Results:**

Patients with comorbidities had lower treatment success rates and higher mortality rates. Diabetes was identified as a significant risk factor for increased TB mortality (OR = 1.9) and mortality from all other causes (OR = 4.5). Similar associations were found for alcoholism (OR = 8.3 and OR = 7.1), immunosuppressive therapy (OR = 5.7 and OR = 5.9), and cancer (OR = 3.4 and OR = 15.4). HIV and tobacco use were associated with an increased risk of mortality from causes other than TB, with odds ratios of 28.6 and 2.2, respectively. The overall treatment success rate in the study population was 88.0%, with 9.2% of patients failing to achieve treatment success and 2.8% dying. Comorbidities such as diabetes, alcoholism, substance addiction, immunosuppressive therapy, cancer, and tobacco use increased the risk of tuberculosis treatment failure.

**Conclusion:**

Patients with comorbidities face a higher risk of unsuccessful treatment outcomes and increased mortality. It is essential to implement integrated management strategies that address both TB and comorbid conditions to improve treatment success rates and reduce mortality.

## 1. Introduction

Tuberculosis (TB) remains a major public health challenge worldwide, with an estimated 10.6 million new cases and 1.6 million deaths in 2021. Tuberculosis is second only to COVID-19 in terms of death from a single infectious agent (1). The burden of TB is not evenly distributed, with the majority of cases occurring in the South-East Asia (45%), Africa (23%), and Western Pacific (18%) regions, as reported by the World Health Organization (WHO) in 2021 (1). The Eastern Mediterranean (8.1%), Americas (2.9%), and Europe (2.2%) accounted for smaller proportions of TB cases. Poland, has made commendable progress in reducing the burden of TB and demonstrated significant strides toward pre-elimination, with a decline in TB incidence from 18 to 9.7 per 100,000 individuals between 2016 and 2021. MDR/RR-TB had been diagnosed in 1.7% bacteriologically confirmed TB cases in Poland (2). Despite the overall decrease in TB cases in Poland and Europe as a whole, there remains a crucial need to enhance our understanding of the factors influencing treatment outcomes and mortality rates among TB patients. The World Health Organization’s EndTB strategy has thoroughly examined various factors that impact the incidence and mortality of TB, with a particular focus on identifying modifiable elements such as the management of comorbidities (3). The relationship between tuberculosis and non-communicable diseases, as well as tuberculosis and other communicable diseases, should be considered in terms of comorbidity, causation, and healthcare. Assessing coexisting diseases is essential to evaluate commonalities, synergies, and challenges for integrating tuberculosis care and control efforts, and to suggest changes in health policies (4). Numerous studies have indicated that comorbidities, including diabetes, HIV, alcoholism, drug addiction, immunosuppressive therapy, cancer, and tobacco use, play a significant role in influencing both TB treatment outcomes and mortality rates (5–8). The risk of exposure, infection, and progression to active tuberculosis is affected by several risk factors, among which coexisting diseases are only one of the risk factors. One should also consider socio-economic and behavioral aspects (9). Socio-economic determinants of health encompass the societal, political, and economic circumstances within which individuals are born, grow, reside, labor, and age (10). All these factors contribute to the complex epidemiology of tuberculosis. However, it is important to note that TB epidemiology is characterized by its heterogeneity (11). The geographical variation in the burden of tuberculosis and associated comorbidities influences the necessity of tailoring medical strategies in different parts of the globe, while also not forgetting to experiences from other geographical regions (4). After identifying diseases coexisting with tuberculosis, the proper strategy for the prevention and management of chronic communicable and non-communicable diseases is an essential element for health systems strengthening, regardless of its geographical location (12). In order to make progress toward TB elimination, it is imperative to gain a better understanding of this heterogeneity and address it accordingly (13, 14). Therefore, conducting epidemiological analyzes at various levels and using different research methods is necessary. A crucial aspect of such assessments is the evaluation of registries maintained at the national level.

However, the impact of comorbidities on TB treatment outcomes in the Polish context has not been extensively investigated. Therefore, the present study aims to bridge this knowledge gap by examining the association between comorbidities and TB treatment outcomes as well as mortality rates among TB patients in Poland. According to the EndTB strategy, expanding knowledge about diseases associated with tuberculosis can allow for better planning of tuberculosis detection and treatment systems, taking into account local conditions.

The objective of this nationwide cohort study was to assess the influence of comorbidities on treatment outcomes and mortality rates in TB patients in Poland.

## 2. Materials and methods

### 2.1. Study design and population

**Figure 1:**
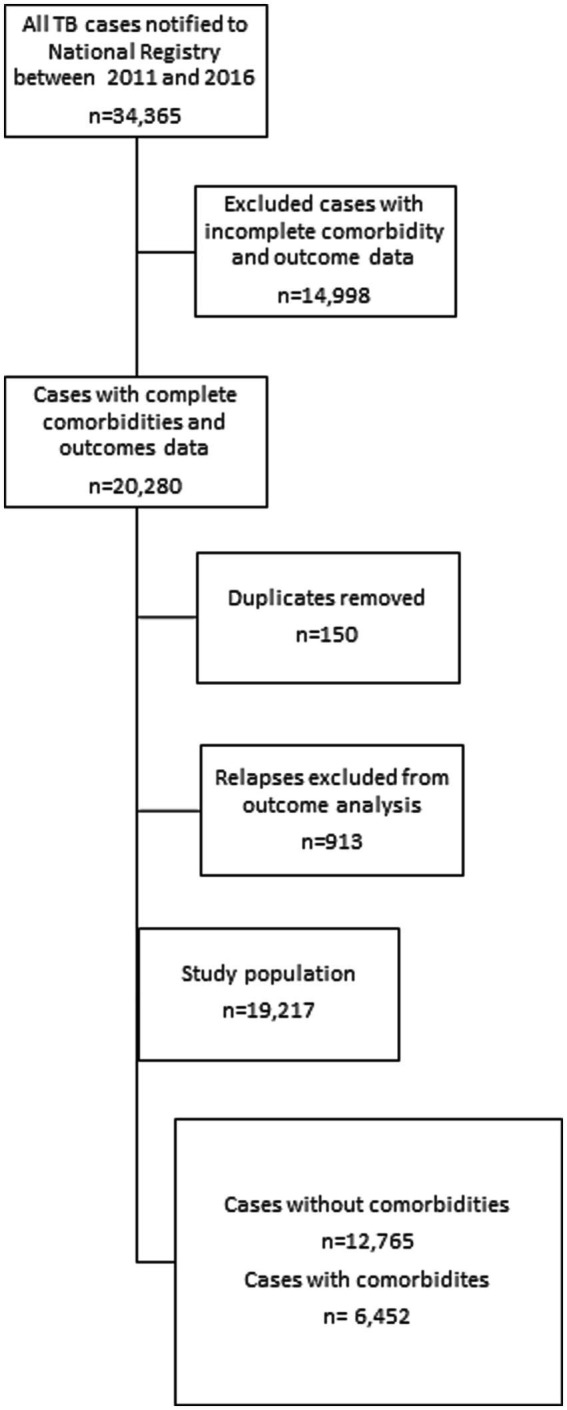
Study flowchart.

This retrospective national cohort study exploited data from the National Tuberculosis Registry (NTR), which is operated by the National Tuberculosis and Lung Diseases Research Institute in Warsaw, Poland. The study population consisted of 34,365 TB patients notified between 2011 and 2016. Patients with missing or incomplete data on comorbidities or treatment outcomes were excluded from the analysis Finally, after the data curation process described above, patients who met the criteria of having confirmed tuberculosis, documented comorbidity, and a recorded treatment outcome were included in the analysis (*N* = 19,217). The study chart is presented in Figure 1.

### 2.2. Data collection

Data on patient characteristics, comorbidities, and treatment outcomes were extracted from the NTR. The presence of comorbidities was assessed based on the International Classification of Diseases, Tenth Revision (ICD-10) codes recorded in the registry. We focused on comorbidities that have been consistently associated with worse TB treatment outcomes in previous studies: diabetes (E10-E14), HIV (B20-B24), alcoholism (F10), drug addiction (F11-F19), immunosuppressive therapy (Z79), cancer (C00-C97), and tobacco use (Z72.0, F17). According to the assumptions adopted by the National Tuberculosis Registry in Poland, the database included comorbidities that were considered as the primary comorbidity by the attending physicians. These categories were also used for further analysis.

### 2.3. Outcome categories

Treatment outcomes were primarily categorized according to the World Health Organization (WHO) definitions, including treatment success (cured or treatment completed), treatment failure, death, loss to follow-up, and not evaluated (15). Monitoring the effectiveness of TB treatment is crucial both in clinical practice and surveillance. Standardized TB treatment outcome definitions have been a significant aspect of WHO policies and national TB surveillance systems. In light of the changes in TB outcome definitions between 2013 and 2022, our analysis relied on WHO recommendations (16), while also maintaining the existing distinction in the Polish TB registry between TB-related deaths and deaths from other causes. Such differentiation can help assess the impact of comorbidities on treatment outcomes. The categories used in the NTR were utilized, including mortality defined as death due to tuberculosis, mortality not connected to tuberculosis, mortality for unknown reasons, patient transferred, and patient still under treatment. Finally, four categories were employed for the analysis. The first category is treatment success, which encompasses the combined categories of “cured” and “treatment completed.” Such a definition of treatment success is a WHO definition, which consists of “The sum of cured and treatment completed” (15).

This serves as the baseline level against which the risks of the other categories are compared. The second category is death due to tuberculosis, which includes cases of individuals who have died as a result of tuberculosis before or during treatment. The third category is death from causes other than tuberculosis, which combines cases of individuals deceased from any cause unrelated to tuberculosis before or during treatment, as well as cases where the cause of death was unknown. The fourth category is labeled as other treatment outcomes, which includes cases of treatment failure, treatment interruption or non-administration, cases where individuals were transferred to another facility, and cases where individuals are still undergoing treatment.

## 3. Statistical analysis

Descriptive statistics were used to summarize the baseline characteristics of the study population, stratified by comorbidity status. The association between comorbidities and TB treatment outcomes was examined using multinomial regression models. There were total 34,364 records for given 5-year period. 913 patients (almost 3% of all patients) had more than one TB episode during the analyzed 5-year period. To ensure the independence of data in the regression model, we decided to analyze only one record (corresponding to the earliest TB episode between 2012 and 2016) for each patient.

We encoded the result of treatment in the following way: “Treatment success”—merged categories “cured” and “ended treatment,” “Death—tuberculosis”—category “death from tuberculosis before and during treatment,” “Death—not tuberculosis”—merged categories “death from other cause before and during treatment” and “death—missing data on the cause of death,” “Other result”—merged categories “treatment failure,” “treatment interrupted or patient not treated,” “patient transferred” and “still under treatment.”

The comorbidity variable contains information about one main comorbidity for given patient and it can be either “none” or diabetes, alcoholism, drug addiction, HIV, immunosuppressive therapy, cancer or tobacco use. Due to missing data in comorbidity variable, the final dataset for the multinomial regression model consists of 19,217 records. We compared individuals without comorbidities (unexposed group) to those with various comorbidities (exposed group) in order to assess the influence of comorbidities on tuberculosis treatment outcomes.

For the final dataset for the regression model, descriptive statistics were presented: mean, standard deviation, minimum, 25% centile, median, 75% centile and maximum for continuous variables; count and percent for categorical variables.

To analyze the influence of main comorbidity on the result of tuberculosis treatment, we chose the multinomial regression model. The outcome variable, the result of treatment, has 4 levels described above. Last three levels can be considered as three different ways of treatment failure. Results of the model are presented in terms of odds ratios (and corresponding 95% confidence intervals and *p*-values) for those three treatment failure categories. They can be interpreted as risks of death from TB, death from different reason or other result of treatment in reference to treatment success, which is the baseline level of the outcome. Our dependent variable in the model is mortality from tuberculosis/other diseases, while comorbidities, relapse, gender, and age are explanatory variables, the independent ones. Baseline level of comorbidity variable is “none,” so the odds ratios can be interpreted as proportions of risk for patients with given comorbidity (e.g., diabetes) and no comorbidities. Model is age and gender adjusted.

Results of the model are graphically presented on forest plots with logarithm scale (due to high odds ratios for certain categories), separately for three categories of treatment failure. All statistical analyzes were conducted using R version 4.2.1 (The R Foundation for Statistical Computing) and a *p-*value < 0.05 was considered statistically significant.

## 4. Results

### 4.1. Characteristics of the study group and comorbidities

**Figure 2:**
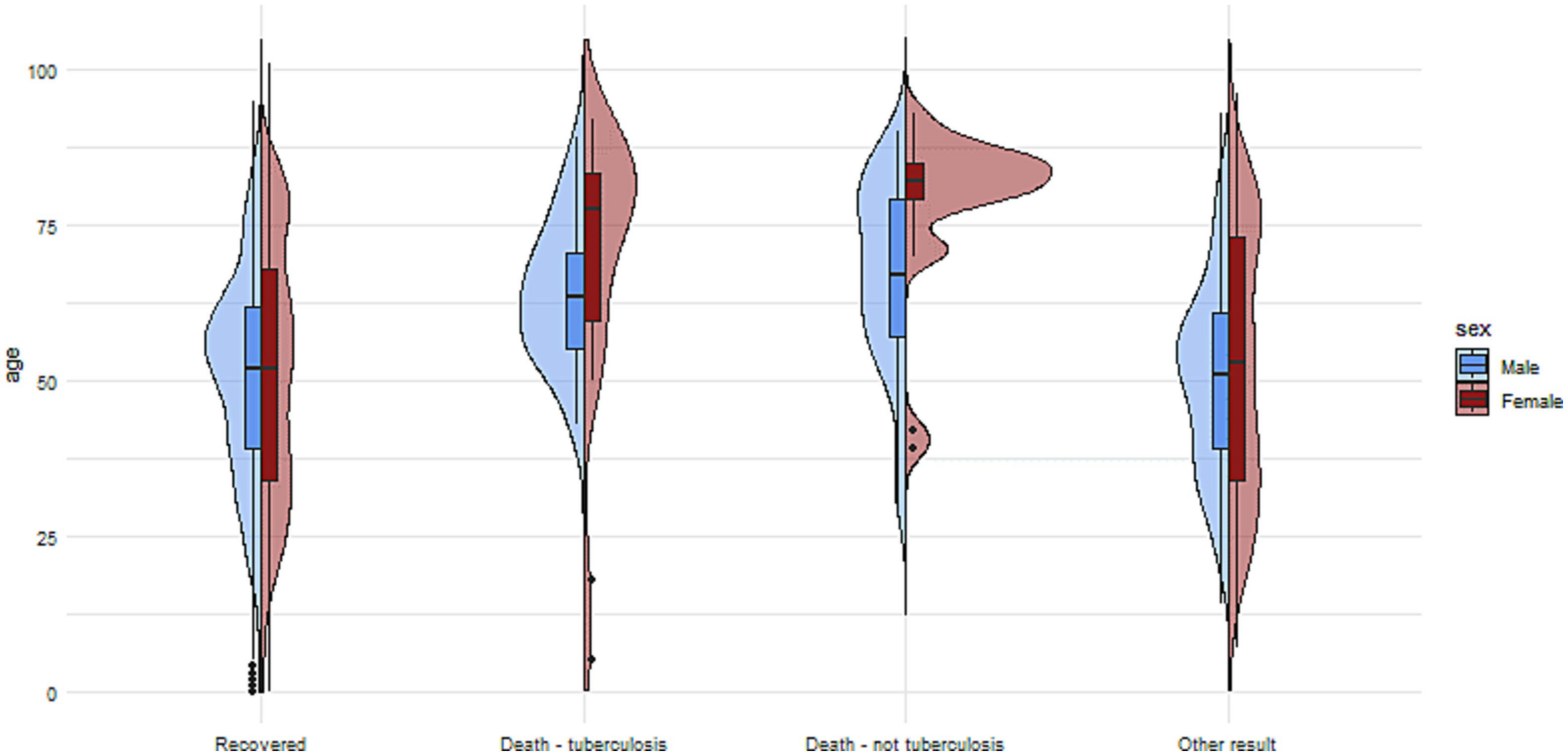
The charts illustrate the age and sex of patients in the group with no reported comorbidities, divided into subgroups based on treatment outcomes.

**Figure 3:**
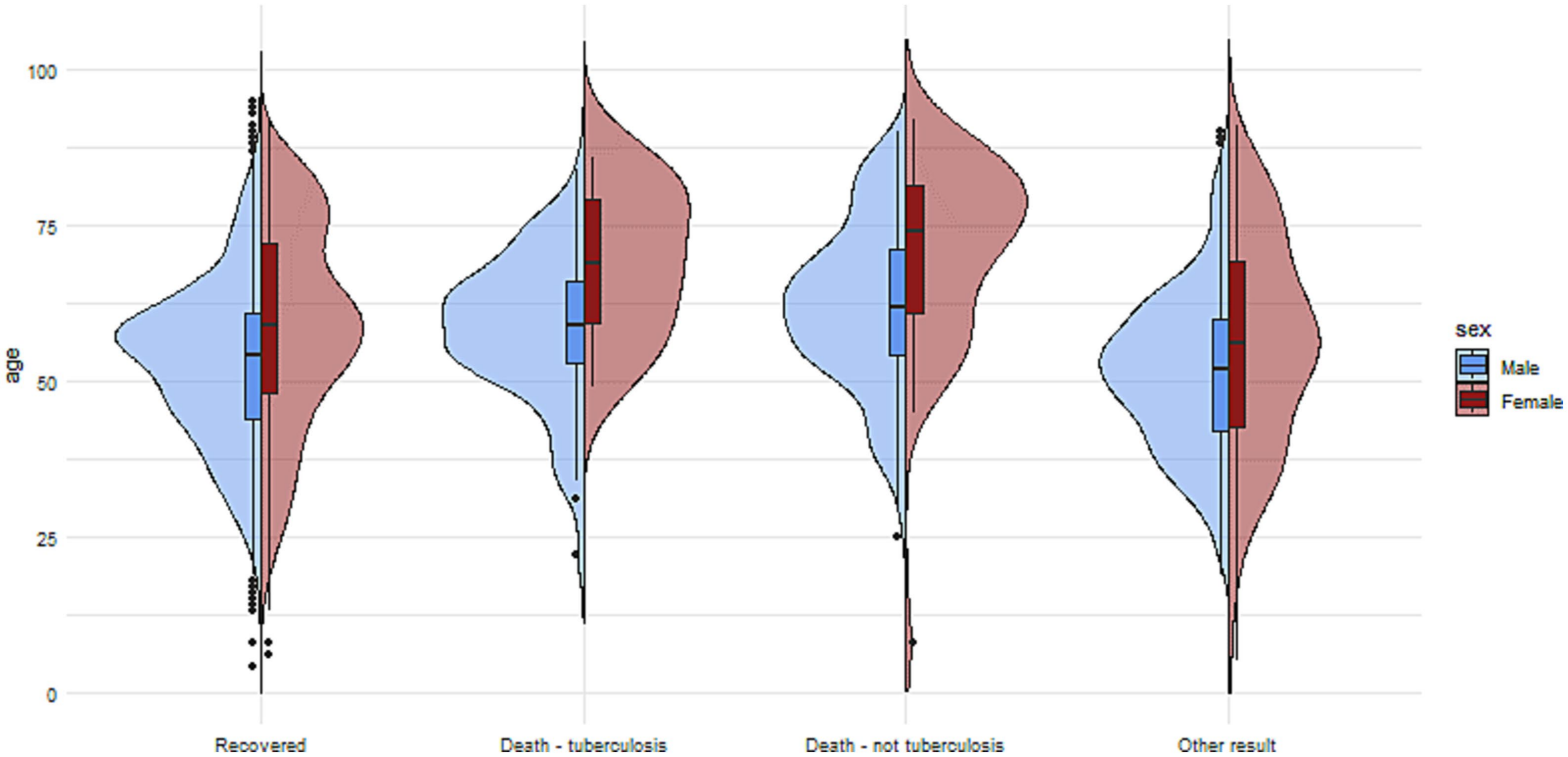
The charts illustrate the age and sex of patients in the group with reported comorbidities, divided into subgroups based on treatment outcomes.

The demographic and clinical data and comorbidities of included patients are shown in Table 1 and Figures 2, 3.

**Table 1:** Baseline characteristics of the study group (*n* = 19,217).

TB registry—descriptive statistics	*n* = 19,217^a^
**Age**	
Mean (SD)	52 (17.4)
Median [IQR]	53.0 [40.0, 63.0]
Range	0.0, 101
**Sex**	
Male	12,838 (67%)
Female	6,379 (33%)
**Year of TB case notification**	
2012	4,756 (25%)
2013	4,240 (22%)
2014	3,731 (19%)
2015	3,185 (17%)
2016	3,305 (17%)
**New case of TB or relapse**	
New case	17,610 (92%)
Relapse	1,607 (8.4%)
**Treatment outcome—original categories**	
Cured	8,096 (42%)
Treatment completed	8,804 (46%)
Death (not tuberculosis)	363 (1.9%)
Treatment failure	17 (<0.1%)
Treatment interrupted or patient not treated	1,165 (6.1%)
Patient transferred	524 (2.7%)
Still under treatment	74 (0.4%)
Death (tuberculosis)	152 (0.8%)
Death (unknown reason)	22 (0.1%)
**Treatment outcome—merged categories**	
Treatment success	16,900 (88%)
Death—tuberculosis	152 (0.8%)
Death—not tuberculosis	385 (2.0%)
Other result	1,780 (9.2%)
**Comorbidities**	
None	12,765 (66%)
Diabetes	1,132 (5.9%)
Alcoholism	2,389 (12%)
Drug addiction	23 (0.1%)
HIV	72 (0.4%)
Immunosuppressive therapy	289 (1.5%)
Cancer	816 (4.2%)
Nicotinism	1,731 (9.0%)
**Clinical classification**	
Pulmonary tuberculosis	17,810 (93%)
Extra pulmonary tuberculosis	1,407 (7%)
Drug-susceptible tuberculosis	12,446 (99%)
Multidrug resistant and rifampicine resistant tuberculosis	115 (0.6%)
Drug resistance not submitted to registry	6,656

a *n* (%).

In the subset of patients included in the multinomial regression model (*n* = 19,217), the mean age was 52 years (SD = 17.4), with a median of 53.0 years (IQR = 40.0–63.0). The age range varied from 0.0 to 101 years. Among these patients, 67% were male (*n* = 12,838), and 33% were female (*n* = 6,379).

The distribution of TB cases across the years in this subset was as follows: 2012 (24%), 2013 (22%), 2014 (19%), 2015 (17%), and 2016 (17%). The majority of cases were new TB cases (92%, *N* = 17,610), while relapse cases accounted for 8% (*n* = 1,607).

Among the patients included in the analysis (*n* = 19,217), the majority of them (88%) achieved a favorable outcome and were classified as “treatment success.” A small proportion (0.8%) died due to tuberculosis, while 2.0% died to causes other than tuberculosis. Additionally, 9.2% of the patients had other treatment outcomes, meaning they did not achieve treatment success.

In terms of comorbidities, 34% of the patients had reported at least one type of primary comorbidities. Among those with documented comorbidities, the most common were diabetes (5.9%), alcoholism (12%), nicotinism (9%), immunosuppressive therapy (1.5%), and cancer (4.2%), HIV (0.4%). Other comorbidities such as drug addiction, were less Figures 1, 2 present the distribution of age and sex among tuberculosis patients with comorbidities and without comorbidities.

In the study group, there were 421 cases in the age range of 0–18 years, which accounts for 2.2% of the total. Descriptive data of pediatric patient subgroup have been presented in Table 2.

**Table 2:** Baseline characteristics of the pediatric subgroup (ages 8–18 years) of the study group (*n* = 421).

TB registry—pediatric population—descriptive statistics	*n* = 421
**Treatment outcome**	
Cured	407 (97%)
Death—tuberculosis	1 (0.2%)
Death—not tuberculosis	1 (0.2%)
Other result	12 (2.9%)
**Comorbidities**	
None	401 (95%)
Diabetes	4 (1.0%)
Alcoholism	1 (0.2%)
Drug addiction	1 (0.2%)
HIV	1 (0.2%)
Immunosuppressive therapy	9 (2.1%)
Cancer	0 (0%)
Nicotinism	4 (1.0%)

### 4.2. Mortality rates

During the study period, 537 [2.79, 95% CI: (2.57–3.04%)] patients died while on TB treatment. 152 [0.79, 95%CI: (0.67–0.93%)] cases were notified as death due to tuberculosis and 385 [2.00, 95%CI: (1.81–2.21%)] as death for all other reason.

In the multinomial regression analysis, the presence of comorbidities was significantly associated with a higher risk of mortality.

Detailed data is presented in Table 3. In the presented table, the results of an analysis examining the association between comorbidities and mortality in tuberculosis patients are shown. The table includes adjusted odds ratios (OR) and 95% confidence intervals (CI) for various comorbidities. The reference category or baseline, represented by the “Intercept” column, corresponds to the group of patients without any comorbidities.

**Table 3:** The impact of comorbidities on the risk of death due to tuberculosis, the risk of death from all other causes excluding tuberculosis, and the risk of outcomes other than treatment success.

	(Intercept)	Diabetes	Alcoholism	Drug addiction	HIV	Immunosuppressive therapy	Cancer	Nicotinism	Relapse	Female	Age
Death—tuberculosis	0 (0, 0.001)***	1.905 (1.031, 3.517)*	8.339 (5.557, 12.515)***	–	6.516 (0.871, 48.743)	5.784 (2.595, 12.891)***	3.368 (1.856, 6.109)***	0.573 (0.207, 1.591)	1.261 (0.771, 2.065)	0.678 (0.453, 1.015)	1.05 (1.037, 1.063)***
Death—not tuberculosis	0 (0, 0.001)***	4.543 (3.203, 6.443)***	7.006 (5.075, 9.671)***	22.472 (4.801, 105.183)***	28.631 (12.285, 66.726)***	5.935 (3.181, 11.073)***	15.379 (11.487, 20.59)***	2.203 (1.413, 3.433)***	0.985 (0.684, 1.418)	0.505 (0.388, 0.657)***	1.058 (1.049, 1.067)***
Other result	0.08 (0.067, 0.096)***	1.702 (1.38, 2.099)***	3.857 (3.407, 4.367)***	3.085 (1.034, 9.21)*	1.83 (0.868, 3.859)	1.798 (1.222, 2.647)**	2.487 (1.99, 3.109)***	1.296 (1.08, 1.555)**	1.342 (1.147, 1.57)***	0.729 (0.647, 0.822)***	0.999 (0.996, 1.003)

Multinomial model—summary (Odds Ratios, 95% confidence intervals, statistical significance).

Statistical significance: ***p < 0.001; **p < 0.01; *p < 0.05.

For the outcome “Death—tuberculosis, “the following comorbidities showed significant associations; for patients with diabetes, the odds of death from tuberculosis were 1.90 times higher compared to the patients without comorbidities. The same for alcoholism—the odds of death from tuberculosis were 8.34 times higher, for immunosuppressive therapy, the odds ratio 5.78 times higher and cancer the odds of death from tuberculosis were 3.37 Age was associated with higher odds ratio of death due to tuberculosis, with 5% higher odds ratio per every year.

For the outcome “Death—not tuberculosis, “similar associations can be observed. For patients with diabetes odds of death from all, other than tuberculosis reasons tuberculosis were 4.54 times higher compared to the patients without comorbidities, alcoholism showed OR 7.01, drug addiction OR 22.47, HIV 28.63, immunosuppressive therapy OR 5.94, cancer 15.38, and nicotine addiction 2.20. In addition older age show significantly higher odds of death compared to the patients without comorbidities.

In comparison of genders, it was found that women had a significant lower likelihood of death with OR (0.505) comparing to man when death for other than tuberculosis reason was analyzed.

### 4.3. Treatment outcomes

Overall, the treatment success rate among the study population was 87.9% [95%CI: (87.5–88.4%)]. 42.1% [95%CI: (41.4–42.8%)] patients were notified as cured and 45.8 [95%CI: (45.1–46.5%)] as a treatment completed. In the group that did not achieve treatment success (i.e., belonged to the category “other”), there were 9.26% [95%CI: (8.86–9.68%)] of all the individuals.

**Figure 4:**
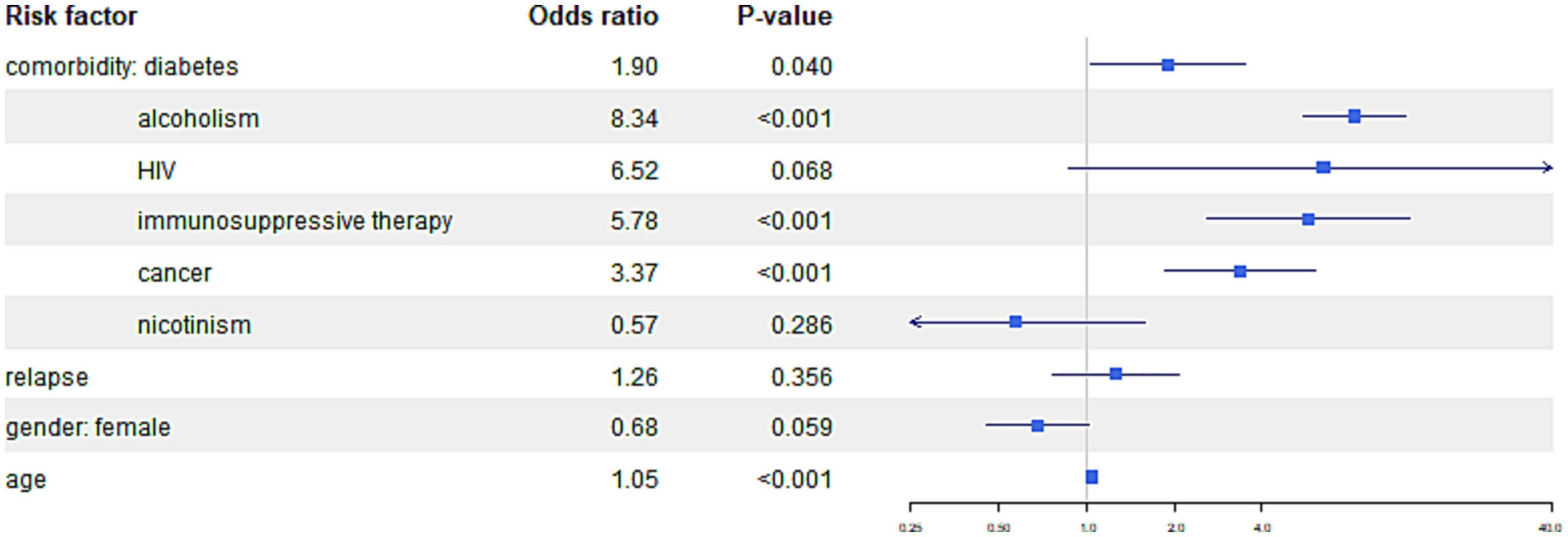
The figure illustrates the risk of mortality from tuberculosis among TB patients with specific comorbidities.

**Figure 5:**
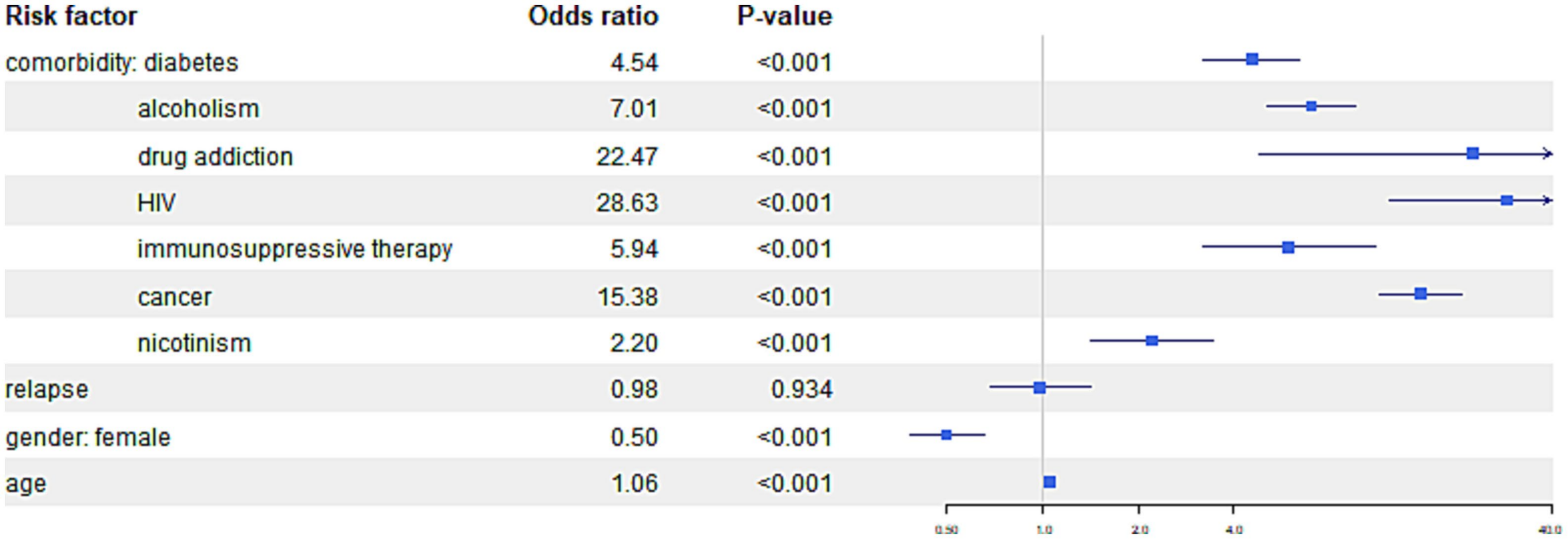
The figure presents the risk of mortality from causes other than tuberculosis among TB patients with specific comorbidities.

**Figure 6:**
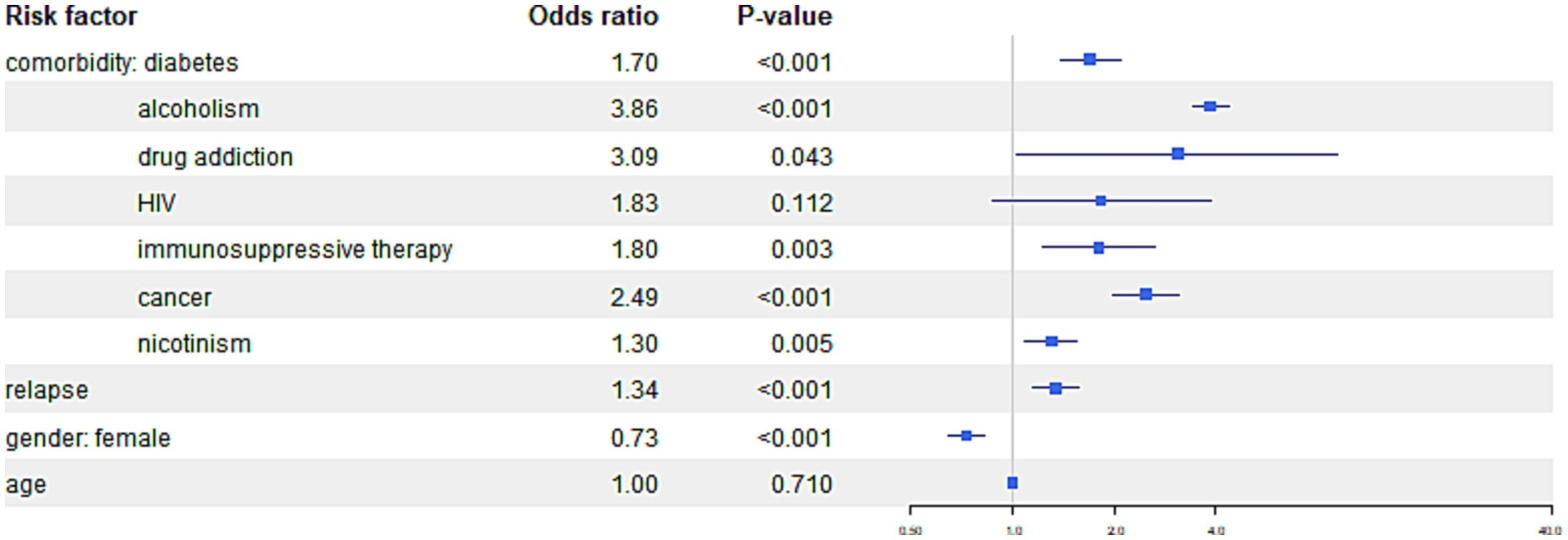
The figure presents the risk of the “other result” outcome category, indicating treatment failure, treatment interruption or patient not treated, patient transfer, and patients still under treatment among TB patients with specific comorbidities.

The comparisons of the impact of comorbidities on different outcomes are presented in Figures 4–6.

**Figure 7:**
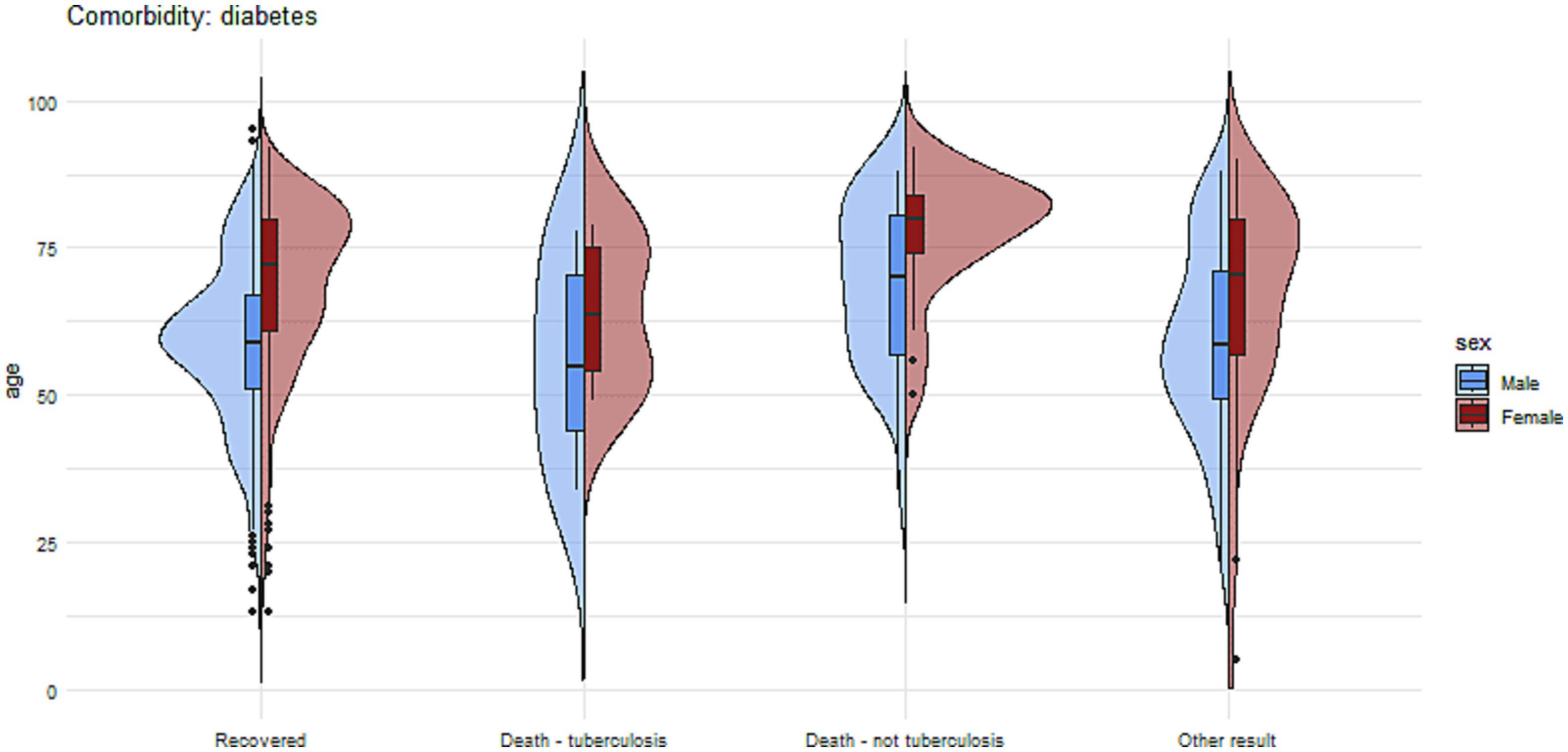
The charts illustrate the age and sex of patients in the group with diabetes mellitus, divided into subgroups based on treatment outcomes.

**Figure 8:**
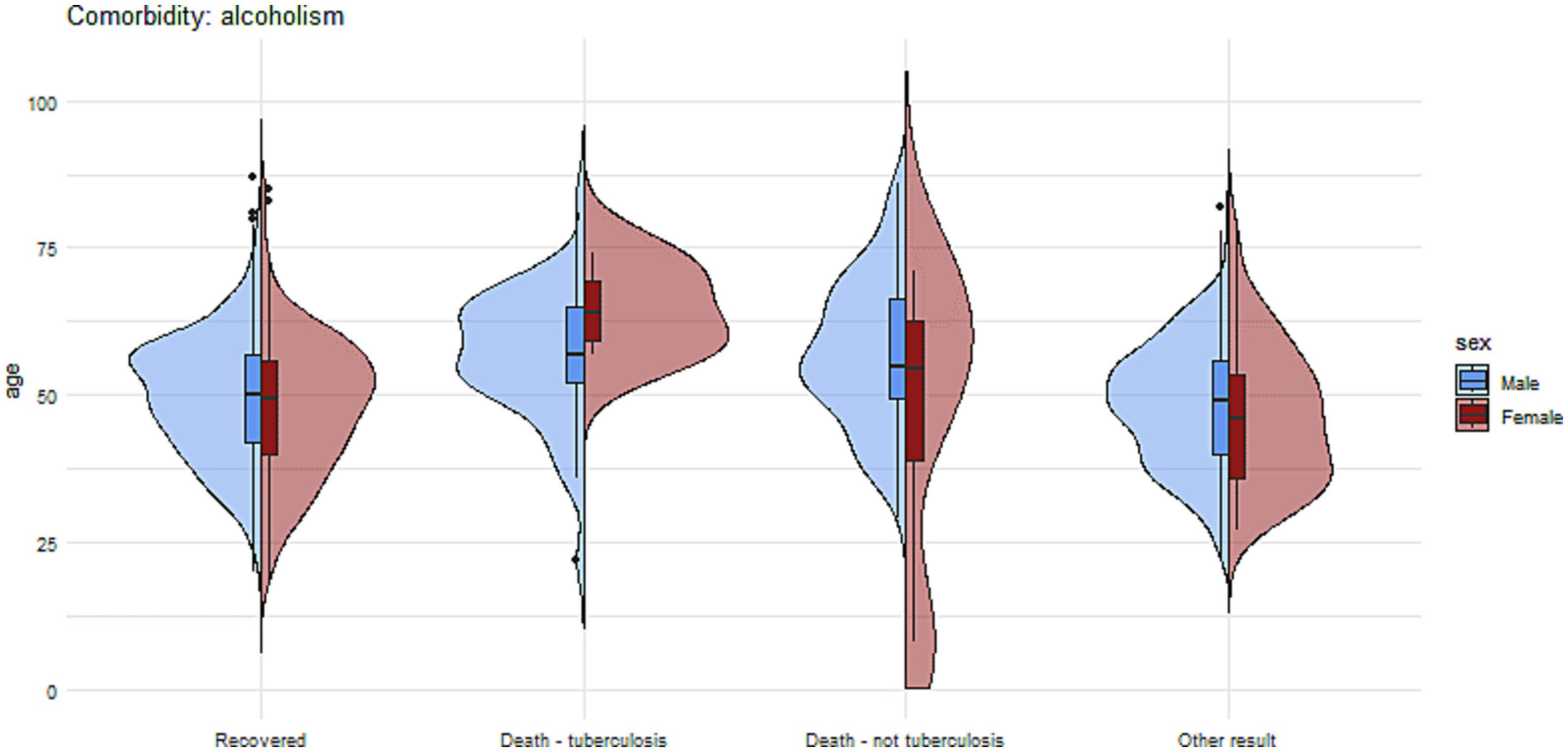
The charts illustrate the age and sex of patients in the group with alcohol addiction, divided into subgroups based on treatment outcomes.

Figures 7, 8 present more detailed information on the age and gender of patients with tuberculosis and comorbidities such as diabetes and alcohol addiction.

In our analysis, the clear influence of gender on the odds ratio of death and the likelihood of treatment success is notable. Women have a 50% lower odds ratio of death from causes other than tuberculosis and a 27% lower odds ratio of achieving an outcome other than treatment success in tuberculosis. In women, there is also a tendency toward a lower odds ratio of death due to tuberculosis, although the result was not statistically significant in this case (Figures 4, 5).

## 5. Discussion

The findings of this study contribute to the growing body of evidence on the influence of comorbidities on TB treatment outcomes and mortality rates. Our results indicate that the presence of comorbidities, as diabetes, HIV, alcoholism, drug addiction, immunosuppressive therapy, and cancer, is associated with lower treatment success rates and higher mortality among TB patients in Poland. These findings are consistent with studies conducted in other countries and settings, highlighting the importance of addressing comorbidities in TB management.

Diabetes has been well-documented as a significant risk factor for TB, as well as an important predictor of adverse treatment outcomes. Both the risk of tuberculosis occurrence in diabetic patients (5, 17) and the impact of diabetes on tuberculosis treatment outcomes were analyzed (18–21). In general, it has been found that the coexistence of diabetes and tuberculosis increases the risk of death, treatment failure, and disease relapse (20). Studies were conducted in multiple regions of the world showing slightly different results. A systematic review and meta-analysis by Gautam in South Asia countries (18) found that TB patients with diabetes had a 1.7 times higher risk of death and a 1.7 times higher risk of TB treatment failure compared to those without diabetes. In Africa, in a study of Faurholt-Jepsen (22), diabetes was associated with a five-fold increased risk of mortality (RR 5.09) among HIV uninfected, and a twofold increase among HIV co-infected patient (RR 2.33). In the WHO European Region, in a study of Sahakyan, 5.8% of TB patients had diabetes and the odds of TB treatment failure was 8.99 times higher in diabetes patients (23). So far, however, there has been a lack of such data regarding the Polish population.

Considering the impact of diabetes on mortality and TB treatment outcomes, it is crucial to acknowledge the variations in diabetes prevalence in active tuberculosis across different regions of the world. In the meta-analysis of Noubiap (24), significant regional differences were observed, with the highest prevalence found in North America and the Caribbean (19.7%), the western Pacific (19.4%), southeast Asia (19.0%), and the Middle East and North Africa (17.5%). These regions had significantly higher prevalence estimates compared to Africa (8.0%), South and Central America (7.7%), and Europe (7.5%).

In our study, 5.9% of TB patients had diabetes. Diabetes increased the risk of tuberculosis-related mortality by 2 times and the risk of all-cause mortality by 4.5 times. Additionally, diabetes significantly reduced the chances of treatment success. These results are generally consistent with studies conducted by other authors, although it is noteworthy that there is a quite low prevalence of diabetes in our tuberculosis patient population but a high risk of deaths in this group. This may be considered in the context of the age of the patients in the studied cohort, with a median age of 53 years, which is slightly higher than in other similar studies. Another element that needs to be taken into account is the possibility of underdiagnosing diabetes in the study population. However, considering the traditional model of tuberculosis treatment in Poland, where the majority of patients initiate treatment in hospitals, this is unlikely.

The factors responsible for the connection between tuberculosis and diabetes are diverse and involve compromised immunity, heightened vulnerability to infection, and diminished response to TB treatment due to potential drug interactions and inadequate glycemic control (25). Unfortunately, with limited registry data, we cannot draw too deep conclusions about the pathophysiology of these processes in our study population.

In our study, we found a strong association between HIV and mortality, with HIV-positive patients having a 22 times higher risk of mortality from causes other than tuberculosis during TB treatment. Similarly, living with the HIV virus tends to elevate the risk of death due to tuberculosis, although this observation did not reach statistical significance in this case (OR = 6.52, *p* = 0.068), what was associated with the low number of death cases in this group. This finding aligns with previous research and global registry data, which demonstrate that individuals infected with HIV are more susceptible to tuberculosis. They face a significantly higher risk—up to 20 times—of developing active TB compared to those without HIV, and they also have a substantially greater risk of treatment failure and death (1, 7, 26–28).

According to recent WHO data, among all incident cases of TB in 2021, 6.7% were people living with HIV. The proportion of people with a new episode of TB who were coinfected with HIV was highest in countries in the WHO African region, exceeding 50% in parts of Southern Africa (1).

We found, that in Poland only 0.4% of tuberculosis patients were infected with HIV in a period of analysis. This prevalence of HIV infection reflects a relatively favorable epidemiological situation in Poland regarding HIV virus incidence (29).

HIV-related immunosuppression increases the risk of TB reactivation, rapid progression, and dissemination, leading to more severe disease and higher mortality rates (30). Furthermore, managing TB-HIV co-infected patients is complex due to drug–drug interactions, overlapping toxicities, and immune reconstitution inflammatory syndrome (31). Drawing conclusions from our study, it is always recommended to recommend co-diagnosis of TB and HIV and strengthen the competencies of pulmonologists and infectious disease doctors in the management of both diseases.

Alcoholism and drug or substance addiction have been recognized as significant factors contributing to unfavorable TB treatment outcomes. These associations can be attributed to a combination of various factors. Firstly, individuals struggling with alcoholism or drug addiction may experience compromised immune systems, making them more susceptible to TB infection and hindering their ability to effectively combat the disease (32). Secondly, the increased risk of reinfection among this vulnerable population further complicates their treatment outcomes (33). Persistent exposure to TB in environments where substance abuse is prevalent can lead to recurring infections, making it challenging to achieve successful treatment outcomes (34). Moreover, the issue of poor treatment adherence among individuals grappling with addiction plays a critical role in the treatment’s effectiveness. Substance abuse can lead to erratic behavior, making it difficult for patients to follow the prescribed TB treatment regimen consistently, leading to treatment failure or drug resistance. Lastly, social marginalization can exacerbate the situation (35). People struggling with alcohol or substance addiction may face stigmatization and lack of support, which can negatively impact their access to proper healthcare and their ability to engage in sustained TB treatment (32, 35, 36). Furthermore, alcohol and drug use disorders may contribute to diagnostic delays, leading to advanced disease at presentation and a higher risk of mortality (37, 38). In our study, which included the entire cohort of tuberculosis patients in Poland, alcoholism was found to be a significant factor associated with treatment failure and increased mortality. Patients with alcoholism had an 8-fold higher risk of death due to tuberculosis, a 7-fold higher risk of death from other causes, and a 3.8-fold higher risk of treatment failure. Similarly, the situation was the same with substance addiction and the risk of death or treatment failure. Patients with substance addictions had a nearly 20-fold increased risk of death from causes other than tuberculosis. We observed a high prevalence of individuals with alcohol addiction, accounting for 12%, and a low prevalence of substance addiction. This reflects the situation in Poland, where alcohol consumption remains relatively high. Our results are generally consistent with studies conducted in other countries, which demonstrate a clear impact of alcohol and substance addiction on mortality and treatment outcomes in tuberculosis patients (38, 39).

Immunosuppressive therapy is another factor that can negatively impact TB treatment outcomes. Patients receiving immunosuppressive drugs, such as corticosteroids or biologic agents, are at an increased risk of TB reactivation and progression to active disease (40, 41). In HIV infection and immunosuppressive therapy, the formation of granulomas in the affected area is altered. As immune suppression progresses, there is a decrease in macrophage activation, resulting in fewer epithelioid and Langhans giant cells. In advanced HIV, the granuloma structure becomes poorly organized, with increased necrosis and an influx of neutrophils, in addition to substantially higher numbers of *M. tuberculosis*. The risk of disease progression increases with the advancement of immune suppression (42).

In our study, there were 1.5% of patients with immunosuppression. This group included individuals who were treated with immunosuppressive drugs or corticosteroids. These individuals had a 5.7-fold higher risk of death due to tuberculosis, a 5.4-fold higher risk of death from other causes, and an 80% higher risk of treatment failure.

In TB patients with concurrent cancer, we noticed a higher risk of deaths from causes other than tuberculosis and an elevated risk of treatment failure compared to tuberculosis patients without cancer. In our registry, deaths from causes other than tuberculosis included all other reasons recorded on the death certificate (e.g., heart attack, pulmonary embolism, etc.) except for tuberculosis. A 15-fold increased risk of death in cancer patients seems understandable in the context of neoplasm disease severity. However, the three-fold increase in the risk of death specifically due to tuberculosis raises some discussion. This is likely associated with the overall effects of the cancer disease and the impact of anticancer treatment on the immune system. Cancer is also linked to an increased number of TB cases, potentially attributed to a combination of factors including compromised immune function, malnutrition, and the toxic effects of chemotherapy (43). Moreover, TB diagnosis and treatment can be more challenging in cancer patients due to atypical presentations, overlapping symptoms, and the need to balance TB treatment with cancer therapy (44).

In our study, 9% of tuberculosis patients were tobacco smokers. However, self-reported tobacco smoking information poses a potential study bias, as objective assessments were not feasible. We would expect higher values, but unfortunately, due to the nature of registry data, objective assessments such as measuring cotinine levels in blood or urine were not feasible. Therefore, we have to rely on the results provided by the physicians who submitted the data to the registry.

The smoking group had a 2.2-fold higher risk of death from causes other than tuberculosis and showed no differences compared to the non-smoking group in terms of death due to tuberculosis. At the same time, tobacco smoking increased the risk of treatment failure by 30%. Our observation regarding the decreased likelihood of treatment success is consistent with previous studies. In a meta-analysis conducted by Wang et al. (45), it was found that cigarette smoking is associated with unfavorable treatment outcomes and delayed conversion to negative smear or culture.

The analysis conducted by Burusie et al. (46) also indicated a negative impact of tobacco smoking on tuberculosis outcomes. However, similar to our study, no direct association between tobacco smoking and tuberculosis-specific mortality was observed. Perhaps, the increased mortality from causes other than tuberculosis among smokers in our group was related to other tobacco-related diseases, such as lung cancer or chronic obstructive pulmonary disease.

The findings of this study have important implications for TB control efforts in Poland and beyond. Based on the WHO “Framework for Collaborative Action on Tuberculosis and Comorbidities” (47), our results support the need for integrated, patient-centered care that addresses both TB and comorbidities. This includes early detection and management of comorbidities, appropriate TB treatment regimens, and close monitoring of patients with comorbid conditions.

Several limitations should be considered when interpreting the results of this study. The study was based on data from a single country, limiting the generalizability of the findings to other settings. However, the national wide cohort and the inclusion of multiple comorbidities contribute to the robustness of the analysis. Future research should focus on investigating the impact of comorbidities on TB treatment outcomes in different populations and settings, as well as evaluating the effectiveness of interventions targeting comorbid conditions in improving TB control efforts.

In conclusion, this study contributes to the growing body of evidence on the influence of comorbidities on TB treatment outcomes and mortality rates in Poland. Our results indicate that the presence of comorbidities, such as diabetes, HIV, alcoholism, drug addiction, immunosuppressive therapy, and cancer, is associated with lower treatment success rates and higher mortality among Polish TB patients.

Our study emphasizes the importance of addressing comorbidities in TB management. For example, implementing smoking cessation programs and measures aimed at limiting alcohol and substance abuse (48).

To effectively address the challenges posed by comorbidities in TB patients, a multifaceted approach is required. Health systems need to prioritize the integration of TB and comorbidity care, ensuring that patients receive comprehensive and coordinated management for their conditions (47, 49).

Furthermore, public health programs should focus on addressing the social determinants of health that contribute to the development and exacerbation of comorbidities in TB patients. This includes addressing factors such as poverty, inadequate housing, and limited access to healthcare services, which can increase the vulnerability of individuals to TB and other health conditions. Targeted interventions aimed at reducing the prevalence of risk factors for comorbidities, such as tobacco and alcohol use, can also play a crucial role in improving TB treatment outcomes and reducing the overall burden of disease.

## Data availability statement

The data analyzed in this study is subject to the following licenses/restrictions: Access to registry data is possible after agreement with the National Tuberculosis and Lung Diseases Institute. Requests to access these datasets should be directed to a.nowinski@igichp.edu.pl.

## Ethics statement

Ethical approval was not required for the study involving humans in accordance with the local legislation and institutional requirements. Written informed consent to participate in this study was not required from the participants or the participants’ legal guardians/next of kin in accordance with the national legislation and the institutional requirements.

## Author contributions

AN: Conceptualization, Investigation, Writing – original draft, Writing – review & editing. SW: Writing – review & editing. MK-K: Conceptualization, Conceptualization, Writing – review & editing.

## Funding

The project was funded by the World Health Organization, EPIC 1 program and by National Institute of TB and Lung Disease.

## Conflict of interest

The authors declare that the research was conducted in the absence of any commercial or financial relationships that could be construed as a potential conflict of interest.

## Publisher’s note

All claims expressed in this article are solely those of the authors and do not necessarily represent those of their affiliated organizations, or those of the publisher, the editors and the reviewers. Any product that may be evaluated in this article, or claim that may be made by its manufacturer, is not guaranteed or endorsed by the publisher.
